# Self-Reported Dyspnea Is Associated with Reduced Health-Related Quality of Life in Quaternary Hospital Workers 1 Year Post Mild COVID-19 Infection

**DOI:** 10.3390/healthcare12242534

**Published:** 2024-12-16

**Authors:** Humberto Batista de Macedo Junior, Mauro Felippe Felix Mediano, Daniel Arthur Barata Kasal

**Affiliations:** 1 Department of Research and Education, National Institute of Cardiology, Ministry of Health, Rio de Janeiro 22240-006, RJ, Brazil; hbmjr0@gmail.com; 2 Evandro Chagas National Institute of Infectious Diseases, Oswaldo Cruz Foundation, Rio de Janeiro 21040-360, RJ, Brazil; 3 Internal Medicine Department, State University of Rio de Janeiro, Rio de Janeiro 20551-030, RJ, Brazil

**Keywords:** COVID-19, health-related quality of life, dyspnea, healthcare worker, physical function

## Abstract

Background/Objectives: The COVID-19 pandemic had significant implications for healthcare workers (HWs), especially those that work in hospitals. This study evaluated health related quality of life (HRQOL) and its relationship with dyspnea approximately one year after COVID-19 infection in HWs. Methods: HWs with previous COVID-19 infections were interviewed, and the EuroQol five-dimensional three-level questionnaire (EQ-5D-3L) with a visual analog scale (VAS) was used to evaluate HRQOL. Self-reported clinical and sociodemographic data were also obtained. Data were stratified by the presence of self-reported dyspnea in the moment of the study interview. The association between self-reported dyspnea and HRQOL was evaluated by regression models, either unadjusted or adjusted for potential confounders (for age and sex, marital status, work category, number of comorbidities, and number of days between diagnosis and evaluation). Results: A total of 109 HWs were interviewed; the median number of days post COVID-19 diagnosis for this group was 400 (IIQ 25–75% 321–428). The majority were women (67.9%); the median age was 44 (IIQ 25–75% 38–52) years. Overall, the median EQ-5D-3L score was 0.79 (IIQ 25–75% 0.74–0.85), and the median VAS score was 80 (IIQ 25–75% 70–90). Self-reported dyspnea was indicated by 22 individuals (20.2%). Self-reported dyspnea was associated with lower EQ-5D-3L and VAS scores, both in adjusted and non-adjusted models. In addition, self-reported dyspnea was associated with more problems in carrying out usual activities in both the non-adjusted and adjusted models (*p* < 0.01). Conclusions: Our results underscore the long-term implications of COVID-19, based on persistent perceptions of self-reported dyspnea and its relationship with HRQOL in HWs. Future studies, with extended follow-up and the employment of cardiopulmonary and mental health testing, may help to elucidate the nature and extent of COVID-19 sequelae.

## 1. Introduction

The COVID-19 pandemic represented one of the most complex challenges to global health over the past hundred years. Worldwide casualties attributable to the disease up until January 2023 totaled over six million, with an estimated global cost of over USD 29 trillion [1]. Hospital workers (HWs) were on the frontlines handling the pandemic, often burdened by close contact with infected patients, high exposure levels, and the demanding nature of their work environments imposed by over-loaded healthcare systems [2]. In addition to the potential consequences of infection on physical health, persistent effects on mental health were described worldwide [3,4]. Long-term health complaints after recovery from the acute phase, referred as long COVID, have been recognized since the first year of the pandemic [5] as an important public health concern. These persistent symptoms affect individuals’ physical, mental, and emotional well-being, with potential impacts on the health-related quality of life (HRQOL) of survivors, including HWs [6,7]. Previous studies have shown that HWs experience greater mental health impacts due to COVID-19 than non-HWs, highlighting the need for continuous mental health assessments [8]. The consequences extend beyond individual health, with potential impacts including decreased workforce productivity, increased demands on healthcare services, and economic burdens on both affected individuals and healthcare systems—an especial critical issue for HWs given their essential role during pandemics and their heightened exposure to the virus [9].

Respiratory symptoms, mainly dyspnea, are frequently reported as a long-term health issue after COVID-19, with complaints months after the infection [10] reported by more than half of patients in some studies [11]. Persistent dyspnea after COVID-19 may result from a combination of factors, including lung damage from the virus, inflammation, and changes in respiratory muscle function, which can significantly affect physical, emotional, and social well-being, ultimately impairing HRQOL [12,13]. However, studies evaluating the long-term consequences of dyspnea on HRQOL in HWs who had COVID-19 are scarce. The evaluation of HRQOL in HWs is crucial, as they directly interact with patients in diverse conditions, often requiring a high level of physical and emotional resilience to provide effective care. Previous studies have demonstrated that low levels of HRQOL are linked to a decline in the quality of care delivered by HWs [14,15]. Therefore, the identification of factors associated with a reduced HRQOL may facilitate the development of strategies to enhance well-being in HWs. In this setting, the present study aimed to evaluate the long-term association of dyspnea and HRQOL in a sample of HWs, about one year after recovery from a mild case of COVID-19.

## 2. Materials and Methods

### 2.1. Study Design and Patients

This is a prospective transversal study including a convenience sample of HWs from the National Institute of Cardiology (Ministry of Health, Rio de Janeiro, Brazil) with molecular diagnosis of COVID-19 established by real-time PCR (rt-PCR). During the COVID-19 pandemic, the National Institute of Cardiology provided testing for HWs who exhibited symptoms or had been in contact with individuals diagnosed with COVID-19. An active search was performed to randomly recruit HWs who had tested positive by phone call or by text message. Inclusion criteria were age ≥ 18 years, with previous positive molecular diagnosis of COVID-19, and currently on duty. Individuals without current working ties to the hospital were excluded. The study was approved by the local Institutional Review Board (IRB) on 26 January 2021 under protocol # CAAE 41494720.2.0000.5272. An informed consent form approved by the IRB was obtained from all participants.

### 2.2. Interview and Clinical Evaluation

On the day of the study interview, anthropometry (height, body weight, and body mass index), and vital signs were obtained by a member of the research team. HRQOL was evaluated using the EuroQol 5-dimensional questionnaire (EQ-5D-3L) with the visual analog scale (VAS). The EQ-5D-3L comprises 5 dimensions: “mobility”, “self-care”, “usual activities”, “pain/discomfort”, and “anxiety/depression”. Each dimension is categorized as causing no problems, moderate problems, or severe problems. The scores on the 5 dimensions can be expressed as an overall utility index (EQ-5D-3L index score) ranging from 0 to 1. Scores based on the VAS can range from 0 (worst state) to 100 (best state) [16]. The EQ-5D-3L is validated for the use in Portuguese for Brazilian individuals [17]. In addition, participants were asked about self-reported dyspnea through a simple question: “Do you experience breathlessness, yes or no”? Participants were specifically inquired about any current complaints of dyspnea during their daily living activities [18]. Clinical and sociodemographic data, including ethnic background and comorbidities, were collected during the same interview based on participants’ self-reports.

### 2.3. Statistical Analysis

Participants were stratified according to the presence of dyspnea at the moment of the study evaluation. Variables were tested for normality using the Shapiro–Wilk normality test. Descriptive statistics consisted of a median with a 25–75th interquartile range for continuous variables and a percentage with absolute frequency for categorical variables. Comparisons of numerical variables based on the presence of dyspnea (yes or no) were conducted using the Mann–Whitney test due to their asymmetric distribution. Comparisons of categorical variables by the presence of dyspnea were analyzed using Pearson’s chi-squared test. The odds ratio (95% confidence intervals [CIs]) for the associations between exposure (dyspnea) and binary outcomes (reports of problems with “usual activities”, “pain/discomfort”, and “anxiety/depression”) were determined by logistic regression using maximum likelihood estimation. The beta coefficients (95% CI) for the associations between exposure (dyspnea) and continuous outcomes (EQ-5D-3L and VAS scores) were determined by generalized linear regression models. The gamma distribution, due to the asymmetric and heteroscedastic nature of the residuals, was employed. Models were fitted without adjustments (unadjusted) and adjusted for potential confounding variables (adjusted model including age, sex, marital status, professional category [administrative, technician, or graduate], number of comorbidities, and time elapsed between COVID-19 diagnosis and the study evaluation [days]). Residual plots for each regression model were visually inspected and did not demonstrate major deviations. Statistical analyses were performed using Stata 17.0. Statistical significance was set at *p* ≤ 0.05.

## 3. Results

### 3.1. Subject Characteristics Stratified by Dyspnea

**Figure 1:**
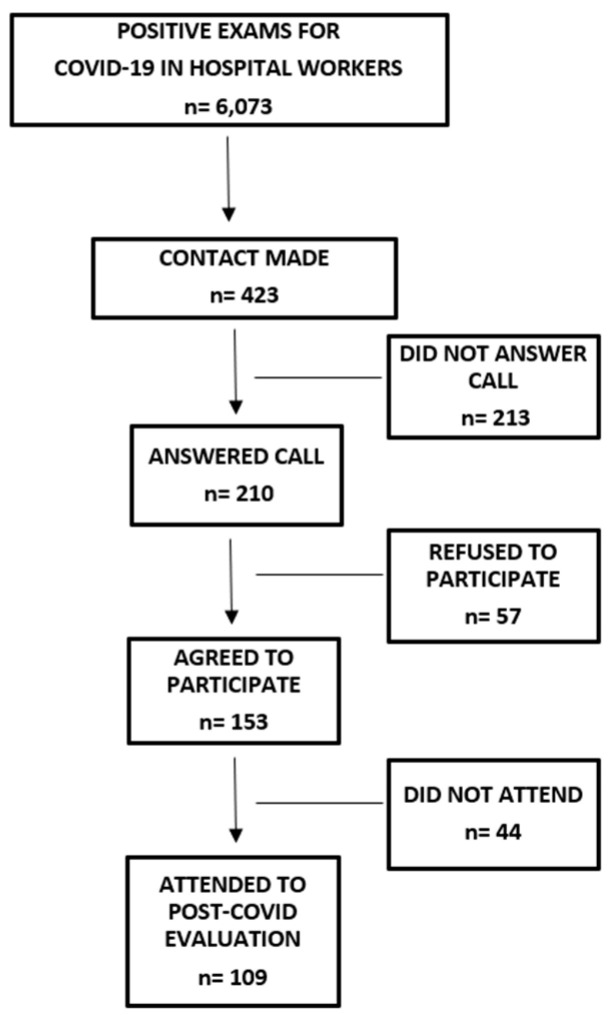
Participant recruitment flowchart.

Between March 2020 and July 2022, 6073 rt-PCR tests performed on HWs were positive for COVID-19. From this number, 423 individuals were randomly invited to take part in the study, and 210 answered the invitation. Of these, 153 individuals agreed to participate, and, finally, 109 performed the evaluation on the scheduled day (Figure 1). All participants had mild cases of COVID-19, with none requiring intensive care hospitalization.

Sociodemographic, clinical, and HRQOL variables stratified by presence of self-reported dyspnea are presented in Table 1. Participants were interviewed at a median of 400 (IIQ 25–75% 321–428) days after diagnosis of COVID-19; the majority of which were women (67.9%) with a median age of 44 (IIQ 25–75% 38–52) years. The median EQ-5D-3L index score was 0.79 (IIQ 25–75% 0.74–0.85), and the median VAS score was 80 (IIQ 25–75% 70–90). The dimension most frequently reported with problems was “anxiety/depression” (57%). Self-reported dyspnea was indicated by 22 individuals (20.2%). Only one participant reported problems in “mobility”, while none reported problems in the “self-care” dimension. Those with dyspnea displayed higher BMIs (*p* = 0.04), lower VAS and EQ-5D-3L index scores (*p* < 0.01), and more frequently reported problems in the dimensions of “mobility” and “usual activities”.

### 3.2. Association Between Dyspnea and HRQOL

The results of the logistic regression (for reports of problems with “usual activities”, “pain/discomfort”, and “anxiety/depression”) or linear regression (for EQ-5D-3L and VAS scores) are presented in Table 2. In both the unadjusted and adjusted models, the presence of self-reported dyspnea was associated with increased reports of problems with “usual activities” (OR 9.68 in the unadjusted model to 14.17 in the adjusted analysis). Additionally, lower EQ-5D-3L scores were observed (β Coefficient −0.04 in both the unadjusted and adjusted models), as well as lower VAS scores (β Coefficient −10.60 in the unadjusted model and −11.43 in the adjusted model). No further associations were observed.

Model adjusted by age, sex, marital status, work category (administrative, technician, or graduate), number of comorbidities, and time elapsed between COVID-19 diagnosis and study evaluation (days).

## 4. Discussion

The main finding of this study was a significant association between the presence of dyspnea and a report of problems in some domains of HRQOL in HWs long after the onset of COVID-19. The long-term effects of COVID-19 and their consequences for daily activities are becoming increasingly acknowledged, particularly as they concern HWs, as the implications for both individuals and healthcare systems are significant. In a recent survey, 94% of the European countries studied identified COVID-19 as an occupational disease [19]. The potential long-term consequences of COVID-19 on physical and mental health may include increased absenteeism, reduced workforce capacity, and heightened healthcare costs, underscoring the need for ongoing monitoring and support for HWs [20,21]. Addressing these issues may help to improve the overall quality of care within healthcare systems facing persistent challenges from the pandemic.

A recent study evaluated HRQOL with the EQ-5D-3L in patients diagnosed with post-COVID syndrome (PCS) who were hospitalized during the acute phase of the disease [6]. The authors found that 75% of patients reported dyspnea, and all domains of the EQ-5D-3L showed higher rates of problems compared to patients without PCS. Of note, in this study only 21.9% of the patients were evaluated 12 months after infection. A recent study evaluating the potential consequences of COVID-19 infection in a sample of 76 patients who were hospitalized found a decrease in the HRQOL index scores among those experiencing dyspnea 12 months after infection, reinforcing the findings observed in our study [22]. In our study, most individuals had mild acute disease symptoms (with only a 5.5% hospitalization rate), and the majority were evaluated at least one year after infection. These findings suggest an extension of the repercussions of dyspnea on HRQOL after acute COVID-19 to less severe patients, and for a long period of time.

Another study conducted in Brazil evaluated HWs using the EQ-5D-3L after acute COVID-19 infection [7]. Six months after infection, there was a worsening in all dimensions of HRQOL compared to before they contracted COVID-19. The age profile and sex distribution (a greater percentage of women) were similar to those of our study. However, no data regarding ethnic background, comorbidities, or professional category were provided; neither was an association between HRQOL with current symptoms investigated. In Brazil, an association between educational level and EQ-5D-3L has been previously demonstrated, with lower education levels associated with reduced index scores [23]. In our study, professional category was used as a proxy of educational level. Interestingly, the association between dyspnea and lower EQ-5D-3L index scores was maintained even after adjustments for potential confounders, including professional category. In this setting, our study extended the implications of COVID-19 for HRQOL in HWs to a more extensive timeframe after acute cases of COVID-19 and was unique in finding an association between dyspnea and HRQOL in this group.

While persistent dyspnea has been consistently demonstrated as long as seven months after infection [24], the mechanisms are not fully understood. In addition, a significant proportion of patients with dyspnea do not present abnormal findings in cardiopulmonary tests [11]. Several explanations are proposed for late respiratory complaints, including viral-induced chronic fatigue syndrome, respiratory muscle dysfunction, and systemic inflammation with the activation of specific T-cells [25,26]. Although the nature of respiratory symptoms presented by participants is outside the scope of the present study, this is an area of interest given their repercussions for HRQOL which endure for over one year after infection.

## 5. Limitations

Some limitations should be acknowledged. The present study was conducted with a relatively small sample of HWs from a single center, recruited through convenience sampling, and therefore may not be representative of the broader population of HWs who have recovered from acute cases of COVID-19. Since studies with convenience samples are more likely to recruit motivated individuals, and that a small sample size generally reduces statistical power, we believe these limitations may have underestimated the associations observed in our study. In addition, although we performed adjusted analyses considering the most important confounders, we cannot exclude the possibility of residual confounding for the relationships between dyspnea and late recovery from COVID-19. Another limitation of our study is its reliance on self-reported measurements of dyspnea, which do not provide information about severity that is typically assessed using the Medical Research Council Dyspnea Scale [27]. While this method is simple and easily integrated into clinical practice, it may be subject to reporting bias and lacks the objective precision of more formal respiratory assessments, increasing the risk for nondifferential misclassification, potentially shifting our results to the null hypothesis. A more detailed evaluation of clinical symptoms of dyspnea, including a cardiopulmonary exercise test, could provide valuable insights into the functional capacity of participants and help better understand the impact of dyspnea on their daily lives.

## 6. Conclusions

The implications of COVID-19 infection even a significant period of time after recovery from acute onset are still incompletely understood. Our results underscore a possible long-term disease effect based on individual perceptions of dyspnea and its relationship with lower self-assessed HRQOL. Future studies, with longer periods of evaluation and which employ cardiopulmonary and mental health testing, may help to elucidate the nature and extent of COVID-19 sequelae.

## Figures and Tables

**Table 1:** Characteristics of participants stratified by self-reported dyspnea (n = 109).

Variables	Total (n = 109)	Self-Reported Dyspnea		
		No (n = 87)	Yes (n = 22)	*p*-Value *
*Sociodemographic*				
Age in years, median (IIQ 25–75%)	44 (38–52)	44 (37–52)	45 (38–53)	0.96
Female, n (%)	74 (67.9)	60 (70.0)	14 (63.6)	0.63
Race, n (%)				
White	56 (51.4)	47 (54.0)	9 (40.9)	
Brown	34 (31.2)	27 (31.0)	7 (31.8)	0.35
Black	19 (17.4)	13 (14.9)	6 (27.3)	
Marital status, n (%)				
Married/stable union	62 (57.9)	53 (61.6)	9 (42.9)	0.12
Other	45 (42.1)	33 (38.4)	12 (57.1)	
Professional category, n (%)				
Administrative	49 (45.0)	36 (41.4)	13 (59.10	
Healthcare technician	23 (21.1)	19 (21.8)	4 (18.2)	0.31
Healthcare graduate	37 (33.9)	32 (36.8)	5 (22.7)	
*Clinical variables*				
Body mass index (kg/m²), median (IIQ 25–75%)	28.1 (24.1–31.5)	26.9 (24.0–31.0)	30.1 (26.4–33.6)	0.04
Hypertension, n (%)	32 (29.3)	25 (28.7)	7 (31.8)	0.78
Diabetes, n (%)	6 (5.5)	5 (5.7)	1 (4.5)	0.82
Obesity, n (%)	42 (38.5)	40 (34.5)	12 (54.6)	0.08
Respiratory disease, n (%)	3 (2.75)	2 (2.3)	1 (4.6)	0.56
Hypothyroidism, n (%)	7 (6.5)	5 (5.8)	2 (9.1)	0.57
Smoking, n (%)	11 (10.1)	11 (12.6)	0	0.08
Comorbidities, n (%)				
1	51 (46.8)	44 (50.6)	7 (31.8)	
2	31 (28.4)	24 (27.6)	7 (31.8)	0.23
3	27 (24.7)	19 (21.8)	8 (36.4)	
COVID-19 vaccination complete, n (%)	94 (86.2)	75 (86.2)	19 (86.4)	0.98
Admission during acute phase, n (%)	6 (5.5)	4 (4.6)	2 (9.1)	0.41
Time between COVID-19 diagnosis and evaluation (days), median (IIQ 25–75%)	400 (321–428)	405 (319–430)	399 (332–422)	0.74
Other symptoms, n (%)				
Chest pain	2 (1.8)	2 (2.3)	0	0.47
Palpitations	3 (2.7)	2 (2.3)	1 (4.6)	0.57
Syncope	0	-	-	-
*Quality of Life*				
Anxiety/depression, n (%)	57 (52.3)	46 (52.9)	11 (50)	0.81
Mobility, n (%)	1 (0.9)	0 (0.0)	1 (4.5)	0.04
Usual activities, n (%)	11 (10.1)	4 (4.6)	7 (31.8)	<0.001
Self-care, n (%)	0 (0.0)	0 (0.0)	0 (0.0)	-
Pain/discomfort, n (%)	49 (45.0)	38 (43.7)	11 (50.0)	0.59
EQ-5D-3L index score, median (IIQ 25–75)	0.79 (0.74–0.85)	0.80 (0.74–0.85)	0.75 (0.74–0.79)	<0.01
VAS score, median (IIQ 25–75)	80 (70–90)	80 (70–90)	70 (70–80)	<0.01

* Mann–Whitney test used for comparison of continuous variables and Person’s chi-squared test used for comparison of categorical variables.

**Table 2:** Association between self-reported dyspnea and health-related quality of life.

Variables	Unadjusted		
	Odds Ratio	*p*-Value	CI 95%
Anxiety/depression	0.89	0.81	0.34 to 2.27
Usual activities	9.68	0.001	2.52 to 37.20
Pain/discomfort	1.29	0.60	0.50 to 3.29
	**β Coefficient**	***p*-Value**	**CI 95%**
EQ-5D-3L index score	−0.04	0.01	−0.06 to −0.01
VAS score	−10.60	0.001	−16.50 to −4.61
	**Adjusted**		
	**Odds Ratio**	***p*-Value**	**CI 95%**
Anxiety/depression	0.98	0.96	0.34 to 2.76
Usual activities	14.17	<0.001	2.71 to 73.98
Pain/discomfort	1.35	0.58	0.47 to 3.89
	**β Coefficient**	***p*-Value**	**CI 95%**
EQ-5D-3L index score	−0.04	0.02	−0.07 to −0.01
VAS score	−11.43	<0.001	−17.53 to −5.33

## Data Availability

Data from this study will be made available upon reasonable request to the corresponding author.
